# Microscopic Colitis: Epidemiology, Pathophysiology, Diagnosis and Current Management—An Update 2013

**DOI:** 10.1155/2013/352718

**Published:** 2013-04-18

**Authors:** Martin Alexander Storr

**Affiliations:** Division of Gastroenterology, Department of Medicine, Ludwig Maximilians University of Munich, Campus Grosshadern, Marchioninistr 15, 81377 Munich, Germany

## Abstract

Microscopic colitis is a common cause of chronic diarrhea. Over the last years the incidence and the prevalence of microscopic colitis are rising and this rise is largely attributed to a rising awareness, and concomitantly an increasing number of diagnoses are made. Patients with microscopic colitis report watery, nonbloody diarrhea of chronic, intermittent, or chronic recurrent course. Following an unremarkable physical examination the diagnosis of microscopic colitis is made by colonoscopy, which shows essentially a normal colonic mucosa. Biopsies taken during the colonoscopy procedure will then finally establish the correct diagnosis. Histological workup can then confirm a diagnosis of microscopic colitis and can distinguish the two distinct histological forms, namely, collagenous colitis and lymphocytic colitis. Presently both forms are diagnosed and treated in the same way; thus the description of the two forms is not of clinical value, though this may change in future. Depending on the patients age and gender 10–30% of patients investigated for chronic diarrhea will be diagnosed with microscopic colitis if biopsies are taken. Microscopic colitis is most common in older patients, especially in female patients and is frequently associated with autoimmune disorders and the consumption of several drugs. This review summarizes the present knowledge of the epidemiology, the pathophysiology, and the diagnosis of microscopic colitis and discusses the former and the present treatment options.

## 1. Introduction

Microscopic colitis is a relatively recent term used for a group of gastrointestinal diseases where chronic watery diarrhea is the leading symptom. The term was coined approximately 30 years ago in a journal case report on a patient with chronic diarrhea. In this specific case report, where the diagnostic workup and the clinical context as well as therapeutic decisions were discussed, the mild inflammatory changes seen by the pathologist in the colonic mucosa where judged as being not-related [1]. The term collagenous colitis is actually a few years older and collagenous colitis now stands for one major form of microscopic colitis [2]. The more recent term, namely, lymphocytic colitis, stands for the other defined major form of microscopic colitis [1]. It is an ongoing matter of debate whether lymphocytic colitis and collagenous colitis really are one disease and should be discussed together as microscopic colitis or whether they are two different diseases that just share some features like clinical presentation and are presently treated in the same way [3–6]. From pathophysiological models these two entities in fact may present two different disorders, and from epidemiological date, where female to male ratio is differently distributed in collagenous colitis and lymphocytic colitis, there are strong arguments that the two diseases are incorrectly grouped together. Additionally from published clinical case reports there is no change of histology from one disease to the other, forming another strong argument that collagenous colitis and lymphocytic colitis may be two distinct diseases. To complicate the matter, just recently a limited number of the so-called paucicellular lymphocytic colitis papers tried to add a third entity under the umbrella of microscopic colitis. Though there seems to be some evidence that paucicellular lymphocytic colitis may exist, there is recent immunohistochemical evidence that it may not be regarded as a member of the microscopic colitis family as some key features of paucicellular lymphocytic colitis, like negative CD25 and FOXP3 immunostaining, clearly distinguishe paucicellular colitis from microscopic colitis [7, 8]. Very recently the term incomplete microscopic colitis (MCi) was introduced, for patients with diarrhea and an increase of cellular infiltrates, that do not fulfill the histological criteria of collagenous colitis or lymphocytic colitis. Whether this selection of patients has to be considered as patients with microscopic colitis or as patients where microscopic colitis is ruled out has to be clarified in future clinical studies [9]. Pathological workup has to rule out the least common of the colitides, namely, eosinophilic colitis. This rare disease has gained increasing awareness during the last years and may still be underestimated but is clearly distinct from microscopic colitis [10–12].

Nowadays we know that the inflammatory changes are closely related to the symptom chronic diarrhea though there are still plenty of unanswered questions like what causes the specific microscopic changes, are the microscopic changes primary pathogenetic changes or secondary changes, and the burning question: what is the exact mechanism that causes diarrhea, when the mucosa is inflamed [13]? We do know that microscopic colitis is an inflammatory disease of the intestine and thus it is regarded as being a new member of the group of inflammatory bowel disease (IBD) [14].

The pathophysiology of microscopic colitis is still unknown but there is strong evidence that microscopic colitis is frequently associated with the use of certain medications and certain systemic disorders (such as autoimmune and rheumatic disease). An invasive endoscopic examination will show a normal colon with macroscopically not inflamed mucosa, and since the typical histological changes in microscopic colitis can be patchy, it is crucial to take biopsies from all colon regions to allow the pathologist to make the diagnosis of microscopic colitis. Presently it is recommended to harvest at least two biopsies from the ascending colon, the transverse colon, the descending colon, the sigmoid colon, and the rectum, in order to have a sensitivity greater than 95%.

Treatment has evolved over the last years and whereas up to 10 years ago antidiarrheal and anti-inflammatory drugs where the mainstay of microscopic colitis treatment, newer clinical trials and meta-analyses have established budesonide as the treatment of first choice of both, collagenous colitis and lymphocytic colitis in the acute and in the long-term treatment.

## 2. Epidemiology, Risk Factors, and Associated Diseases of Microscopic Colitis

Following its first description in the early 80, microscopic colitis was felt to be a rare disease [1, 2, 15–18]. Over the last thirty years we have learned the opposite, that microscopic colitis is a common disease with high incidences and very high prevalence. Early epidemiologic data extracted mainly from retrospective studies that were performed in Sweden showed rising incidences from the years 1983–1998, whereas in the early years of the analyses only scattered and sporadic cases of collagenous colitis and 10 years later also lymphocytic colitis were reported, in the latter years incidences of collagenous colitis and lymphocytic colitis ranged from 3–5 per 10000 [19–22]. What we learned from these studies is that microscopic colitis is a disease of mainly older people, especially of older female patients and this holds true for collagenous colitis and for lymphocytic colitis [23]. Newer epidemiologic studies done in this century confirmed these high incidence numbers showing that actual incidence and prevalence numbers are higher than initially thought and are still able to show rising incidences, though the rise is far less pronounced than before. Most recent north American studies show incidence rates of 7.1 per 100,000 person-years for collagenous colitis and 12.6 per 100,000 person-years for lymphocytic colitis, respectively [24]. The overall prevalence for microscopic colitis was reported being 103.0 per 100,000 persons and splits up into 39.3 per 100,000 persons for collagenous colitis and 63.7 per 100,000 persons for lymphocytic colitis. Both incidence and prevalence rates of microscopic colitis approach those of the classical inflammatory bowel diseases like ulcerative colitis and Crohn's disease [21, 25]. Newer epidemiological studies confirm these high incidences and prevalences also in the European population and in the Asian population and the incidence rates and the prevalence rates for collagenous colitis in the western world seem to level out, whereas the incidence rates for lymphocytic colitis are still rising [26–31].

For a long time it was a matter of debate what drives the rising incidence and a recent study identified that clearly awareness of the disease and practice style of the endoscopists and the pathologists involved are the major drivers for the still rising incidence [32]. The study identified that especially endoscopists with an academic practice style, compared to a private practice setting, were more likely to make a diagnosis of microscopic colitis. Interestingly it was the endoscopists with lower annual endoscopy volumes and physicians with a gastroenterology, compared to an internal medicine or surgery background, performing the colonoscopies who had the highest diagnostic yield for microscopic colitis [32].

The newest clinical studies suggest that at least for collagenous colitis the incidence rates have now leveled out whereas the incidence rates for lymphocytic colitis are still rising [31]. Whether this is just an epiphenomenon of an awareness-detection bias or whether this indicates a still rising true incidence, maybe driven by an increase of a pathological factor (e.g., drug use, environmental factor, and nutritional factor), is a matter of debate and has to be clarified in prospective clinical trials rather than in retrospective or observational studies. With awareness being a recognized driver for the rising incidence rates, recent and forthcoming awareness initiatives may help to increase microscopic detection rates even further [13, 33, 34].

Established risk factors for microscopic colitis are female gender [21, 35, 36], higher age [21, 35, 36], concomitant autoimmune diseases such as thyroid disease or celiac disease [37–39], a past or current diagnosis of malignancy [40, 41], and a history of solid organ transplant [42].

Female gender is a major risk factor and this gender preference is somewhat more pronounced in collagenous colitis [21, 24, 35, 36, 43, 44]. The reasons for this gender distribution are unknown and possible contributions of hormonal alterations or an ascertainment bias in women remain speculative. In population-based studies the female to male ratio ranges from 4.4–7.9 to one for collagenous colitis and only from 1.8–5.0 to one for lymphocytic colitis [20, 21, 24, 35, 36, 40]. Whether this gender difference is due to a reporting bias in epidemiological studies of small numbers yet has to be determined. Talking about gender differences, it is worth mentioning that, in one report, patients that got pregnant after a diagnosis of microscopic colitis lost their clinical symptoms of the microscopic colitis. This loss of symptoms was sustainable and was also evident after child birth. Though other features were not tested, this observation suggests that hormone status may play a role in the pathophysiology of microscopic colitis and warrants further investigation [19].

Numerous studies established that the incidence of microscopic colitis increases substantially with advancing age [21, 35, 36] and the mean age when a diagnosis of microscopic colitis is made lies in the fifth and sixth decades. The reasons for this age distribution are unknown. It has to be kept in mind that this age distribution may be biased, since we are less likely to perform colonoscopies with or without biopsies in younger adults or pediatric patients, and may thus underestimate incidences in younger populations [45]. Prospective clinical trials are needed to establish incidences in younger populations.

30–50% of patients with microscopic colitis have at least one concomitant autoimmune disease. In 10–20% there is an association with thyroid disease and this was shown in early smaller and latter larger cohorts in different populations [21, 37, 38, 40]. Celiac disease is reported to be associated in 5–25% of patients with microscopic colitis and the association with autoimmune disease is stronger with collagenous colitis [22, 38, 40, 44]. Association with malignant disease is less pronounced, but still up to 10% of patients with microscopic colitis have been diagnosed with some form of malignant disease [40]. In patients that received solid organ transplantation, incidence rate of microscopic colitis is approximately 50-fold higher than in the general population [42]. Whether this is due to the disease that led to organ failure and transplantation or more likely due to the immunosuppressant or concomitant medication is not studied yet. Frequently rheumatic disease is listed in the list of associations but it remains unclear whether the disease or the medication or both contribute to this association

Recent casuistic report found juvenile spondylarthritis or the SAPHO syndrome to be associated with microscopic colitis, but other than this, little is known about disease associations in the pediatric population [46, 47].

Strong associations were furthermore reported for microscopic colitis and certain drugs, and the possible causative nature of these associations is a matter of ongoing discussions and study activity. Whereas retrospective data show strong associations, prospective data is still missing and will be soon available. Strong associations were reported for specific NSAIDs, PPIs, SSRIs, beta blockers, statins, and bisphosphonates [19, 48–50]. Interestingly in patients with collagenous colitis, NSAIDs and SSRIs are more commonly used, while in patients with lymphocytic colitis, SSRIs, beta blockers, statins, and bisphosphonates are more commonly used [49, 51–53]. Whether these are just random discrepancies or true differences with different pathophysiologies is unclear, but due to the major impact and consequences such differences would have, further research on these associations seems imperative. For NSAIDs there are good reports that symptoms and histological changes improve with cessation and return with reintroduction giving strong arguments to the notion that these drugs are of major relevance in the pathophysiology of microscopic colitis [54]. A very recent study identified that PPI use increases the number of intraepithelial lymphocytes even without causing diarrhea. This is inasmuch of importance as many authors discuss the association of PPIs and microscopic colitis as being irrelevant since diarrhea is a possible side effect of PPIs and thus may lead to increased diagnostic and in consequence to increased diagnosis of microscopic colitis. Whether this means that intraepithelial lymphocytes are increased before symptom onset or that increased lymphocytes do not cause symptoms in some patients is not clear yet and awaits to be clarified in prospective studies [55]. From present literature it has to be assumed that PPIs are associated with microscopic colitis and this holds true for all known PPIs, though the evidence for such an association seems to be best established for lansoprazole. Though drug disease association plays a strong role in patients with microscopic colitis, considering the large group of drug users and the relatively low incidence of microscopic colitis, drug-induced cases of microscopic colitis are most likely the result of a idiosyncratic reaction [56].

There are numerous reports on other drug uses being associated with microscopic colitis and these associations were reported in individual patients or in small uncontrolled case series. These associations show up for microscopic colitis or the different entities collagenous colitis and lymphocytic colitis. It is presently unclear what these sporadic associations mean and whether these associations mean anything at all or whether they are just random associations. From a 2013 perspective it seems unlikely that these associations are just random since the numbers of patients on which these observations are made are reportedly high. For all the drugs that seem to be associated with microscopic colitis it is always worth to pause these medications if possible and see whether symptoms of the patients improve. If symptoms improve the association may be regarded as relevant, and termination of the harmful medication and replacement with alternative drugs should be recommended. Therefore this review gives a comprehensive list of drugs, even the rare ones implicated to be involved in microscopic colitis (Table 1). Overall it is worth noticing that lymphocytic colitis seems to be associated with drug use more often compared to collagenous colitis and this observation is worth being studied further as it may be one confounder why the incidence of lymphocytic colitis is still rising. Recently duloxetine was reported to induce lymphocytic colitis [57]. In this context it is worth mentioning that not every drug that is associated with a colitis results in microscopic colitis as, for example, for mycophenolate associated colitis, histological changes are clearly distinct from changes seen in microscopic colitis [58].

## 3. Etiology

The etiology of microscopic is most likely multifactorial with a mucosal inflammatory response to yet not specified noxious luminal agent occurring in a predisposed host. The noxious luminal agent may be a single one, or multiple ones summing up to an individual threshold.

Technically microscopic colitis is an inflammatory bowel disease (IBD) and the disease shares a number of etiological aspects with the so-called classical inflammatory bowel diseases like Crohn's disease and ulcerative colitis. Amongst the possible predisposing and/or contributing factors for microscopic colitis, genetic factors and intraluminal noxious factors are best studied.

Though a limited number of familial clusters of microscopic colitis have been reported, there is only minimal evidence of a genetic component within the etiology of microscopic colitis. And this holds true for collagenous colitis and lymphocytic colitis. All reported so-called family clusters are very small and comprise a maximum of 2 reported family members. In contrast there is evidence of a predisposition of sensitivity to gastrointestinal inflammatory insults in patients with microscopic colitis since up to 12% of patients with microscopic colitis have a family history of celiac disease or even inflammatory bowel disease [22]. The meaning of the association between HLA-DQ2, DQ1, DQ3, and microscopic colitis and the high prevalence of a TNF*α* gene polymorphisms in patients with microscopic colitis deserves further attention as it may lead to a discovery of a hereditary component of microscopic colitis of presently unknown penetration [59]. Furthermore metalloproteinase-9 gene variations have been reported to be associated with collagenous colitis [60] but the meaning of all the presently reported genetic associations is poorly understood and the respective research is presently not driven by hypotheses rather than by incidental observations or genetic screening.

Very strong evidence exists for an autoimmune basis to the development of both collagenous colitis and lymphocytic colitis. The association of microscopic colitis with autoimmune-based disorders such as celiac, thyroid disease, and rheumatoid arthritis, as well as the female preponderance, supports the notion that both forms of microscopic colitis have a strong association with autoimmune diseases and may well be an autoimmune disorder themselves. But to date no specific autoantibody has been identified as being diagnostic for or being associated with collagenous colitis or lymphocytic colitis [37]. It is though known that microscopic colitis can be found together with various autoantibodies and phenotypes like HLA DR3 phenotype, though these associations are not strong enough to be regarded as being diagnostically relevant or useful, nor do we know what these associations mean.

Luminal factors of whatever kind seem to play an important role in the pathogenesis of microscopic colitis. Numerous drugs were reported to have a high or at least intermediate probability of causality in microscopic colitis and the drugs found to have high or intermediate associations with microscopic colitis are listed in Table 1. Interestingly, drug association profiles for collagenous colitis and lymphocytic colitis are different, and for lymphocytic colitis the association with certain drugs seem to be stronger than that for collagenous colitis. This once again suggests that collagenous colitis and lymphocytic colitis may be two distinct diseases and do not share a common pathophysiology. The drugs listed as being associated with microscopic colitis should be discontinued when microscopic colitis is diagnosed and drug use is confirmed as this may result in an immediate resolution of the symptoms reported by the respective patient [54]. Especially for NSAIDs, PPIs, and antidepressant or antipsychotic drugs, where the probability is high that symptoms may be caused or at least promoted by the respective drug, the drug should be timely discontinued.

Other luminal factors like infectious or even toxic agents are supported by studies that found either onset of microscopic colitis following a gastrointestinal infection or improvement of symptoms with the initiation of antibiotics in the context of a proven or suspected gastrointestinal infection [19]. *Yesinia species* [61], *Clostridium difficile* [62], and *Campylobacter species* [63] were suggested in published case reports to cause microscopic colitis, though interpreting these observations in the context of current knowledge, it is most likely that these cases are of sporadic nature. In some small retrospective case series, bile acid malabsorption has been found in up to 60% of patients with lymphocytic and up to 44% of patients with collagenous colitis supporting the notion that microscopic colitis may at least in some patients be caused by bile acid malabsorption. Whether bile acid malabsorption is causative or not remains questionable as later studies were unable to confirm these observations [64]. Still, this may direct therapeutic decisions and especially in patients with a cholecystectomy, a bile acid directed treatment should be considered.

Basic science is still in its infancy, when it comes to studying microscopic colitis and possible causes, drivers, mechanisms, or even pathophysiological models. A recent benchside study employing sigmoid tissues from patients with collagenous colitis and lymphocytic colitis was able to identify that sodium transport and epithelial barrier function are disturbed in patients with microscopic colitis [65]. Unfortunately it remains unclear whether these reported changes are of causal nature, of transient nature, or a consequence of the underlying microscopic colitis. But even though these descriptive studies at least initiate a scientific discussion on what may be mechanisms underlying or involved in the development and the resolution of microscopic colitis, these small mechanistic studies have to be carefully taken since such studies are highly artificial in the techniques they use and therefore the results are most likely influenced not only by numerous circumstances like laboratory procedures and protocols but also by patients drug use, patients age, and even patients nutritional status. Thus such information has to be considered as hypotheses generating information that hopefully guides future prospective studies that help us to understand the mechanisms involved in pathogenesis, maintenance, and resolution of microscopic colitis and symptoms associated with the histological changes.

Using molecular techniques it was reported that in patients with microscopic colitis increased IFN-*γ*, TNF-*α*, and IL-1*β* levels suggest a Th1 cytokine profile being involved in the inflammatory process [65]. It is presently not fully understood what the differences in mucosal lymphocyte subsets, seen in patients with collagenous colitis and lymphocytic colitis, mean [66]. This information may help us to understand the inflammatory mechanisms involved and it may be useful for future therapeutic approaches.

Environmental factors may play a crucial role in the etiology of microscopic colitis, though other than cigarette smoking presently no other such factor is confirmed. For both collagenous colitis and lymphocytic colitis cigarette smoking is more prevalent compared to subjects without microscopic colitis and first reports suggest that lung cancer is associated with microscopic colitis [41, 67–69]. The odds ratio for lymphocytic colitis and smoking (OR, 3.8) is higher than for collagenous colitis and smoking (OR, 2.4), though this difference was presently calculated on a small cohort of 120 patients with collagenous colitis, 70 patients with lymphocytic colitis, and 128 controls and thus has to be verified in larger groups of patients [70]. Interestingly, it was additionally shown that microscopic colitis occurs roughly 10 years earlier when the respective person is an active smoker, stressing the relevance of cigarette smoking to the pathophysiology of microscopic colitis [68]. Beyond the strong results from association studies it would be of great impact to learn whether cessation of smoking would cure microscopic colitis or at least be beneficial to the patients symptoms, and a prospective clinical trial answering this seems worthwhile.

In addition to the inflammatory component in the pathophysiology of microscopic colitis, there may be an additional neuronal component to pathophysiology. A recent study identified increased chromogranin A, chromogranin B, and secretoneurin levels in feces of patients with collagenous colitis compared to relevant control groups. These observations may point to a neurogenic involvement in microscopic colitis and additionally these stool markers are suggested to be helpful in discriminating microscopic colitis from irritable bowel syndrome or classical inflammatory bowel disease [71].

## 4. Clinical Presentation

Collagenous colitis and lymphocytic colitis present with very similar symptoms and from a clinical perspective there is no specific symptom or clinical feature that allows discriminating one or the other. Thus the differentiation between the two entities is made by histology only.

The typical clinical presentation involves chronic (either recurrent or intermittent) relapsing watery, nonbloody diarrhea [72, 73]. Only a minority of patients present with an acute onset of their symptoms. Though diarrhea can be moderate to severe in some patients, complications such as changes in electrolyte levels or dehydration are extremely rare.

In contrast to irritable bowel syndrome (IBS) symptoms in patients with microscopic colitis can include nocturnal bowel motions, arthralgias, and fecal incontinence. Other associated symptoms like mild abdominal pain, fatigue, and slight weight loss are similar to the symptoms of patients with IBS and make the diseases difficult to be diagnosed clinically [74]. A recent prospective study on 120 patients identified that clinical symptomatology is inefficient in distinguishing patients with IBS from patients with microscopic colitis and suggested that a colonoscopy is imperative, latest when antibiotic treatment does not relieve the patients symptoms [6, 75]. Similarly to the symptomatic overlap between IBS and microscopic colitis, there is substantial symptom overlap between patients with small intestinal bacterial overgrowth (SIBO) and microscopic colitis. A recent prospective study performed in patients previously diagnosed with IBS suggested that a colonoscopy with biopsies is imperative in these patients and even a routine glucose H2 breath test should be considered, at least when microscopic colitis is ruled out [74]. The majority of patients with microscopic colitis but not all would fulfill the diagnostic criteria for IBS. Following a study from Sweden recruiting patients from 2002 to 2010, 55% of patients with microscopic colitis fulfilled ROME III criteria of IBS. It is worth mentioning, that this study identified that if patients with microscopic colitis fulfill ROME III criteria, they are according to this study more likely to have severe symptoms and have worse psychological well-being, compared to patients with microscopic colitis that do not fulfill ROME III criteria [76].

Most cases of microscopic colitis are self-limiting, with symptoms of an episode lasting for a few months. Other patients are symptomatic for numerous years with either a relapsing or a continuous pattern. For affected patients it is important to know that there is no increased risk of colorectal cancer [41, 72, 77] or development of other inflammatory bowel disease like Crohn's disease or ulcerative colitis [69]. There are case reports on spontaneous and colonoscopy induced colonic perforations in patients with microscopic colitis [78, 79]. Due to the scattered number of reports it remains unknown whether this is a true or a random association. More recent endoscopic reports suggest that mucosal tears that can be found variably in patients with microscopic colitis may be the reason for such perforations [80–84].

## 5. Diagnosis

The diagnosis of MC is made based on normal or minimally nonspecific endoscopic findings with biopsies showing histopathologic findings consistent with a diagnosis of either collagenous colitis or lymphocytic colitis.

When making a diagnosis of microscopic colitis, the first step includes a thorough history with particular attention to the differential diagnoses like IBS, IBD, and infectious colitis as well as to risk factors and diseases associated with microscopic colitis. Radiographic and laboratory tests may be helpful to rule out differential diagnoses but are typically unremarkable with microscopic colitis. There is no increased risk in patients with microscopic colitis undergoing diagnostic procedures and talking about risks; complicated cases like spontaneous perforations are rarely reported [85].

The next step includes a colonoscopy that usually shows a macroscopically normal mucosa. Recent publications list non-specific changes such as abnormal vascular markings, erythema, or mucosal edema but their meanings in the context of the disease remain unclear. It is important that biopsies are taken throughout the colon, as they are needed to make a diagnosis of microscopic colitis. Both collagenous colitis and lymphocytic colitis show a lymphocytic infiltration of the lamina propria and the colonic epithelium [86]. Collagenous can be clearly differentiated from lymphocytic colitis by the presence of a typical, marked thickening of the subepithelial collagen layer [1, 16, 86–88]. The typical histopathological features of collagenous colitis and lymphocytic colitis are listed in Table 2.

One important question is how many biopsies need to be taken and how many biopsies are needed to confirm or rule out microscopic colitis. Numerous studies showed that the microscopic lesions can be skipped, and therefore random colonic biopsies should be taken [89]. Most recent studies show that biopsies of the rectosigmoid colon alone are insufficient to rule out microscopic colitis [90, 91]. Since up to 40% of cases will be missed with biopsies taken from the rectosigmoid region alone, a full colonoscopy with biopsies taken from every region is recommended [23, 32, 92]. It is well accepted that expertise is needed to make a histological diagnosis of microscopic colitis and thus sending the biopsies to a specially trained pathologist is recommended [32].

Novel tools may help us with making the diagnosis of microscopic colitis in future and a recent report on confocal laser endomicroscopy guiding the diagnosis of microscopic colitis is such an attempt [93]. It is presently not clear whether such new diagnostic tools may change the approach and/or the detection rates of the disease as these new tools are not widely used.

## 6. Treatment

Prior to any drug treatment a medication history should be taken and potentially precipitating medications should be stopped. Furthermore associated conditions like celiac disease should be appropriately managed. In some patients dietary restrictions (e.g., avoiding caffeine or lactose) might be helpful, especially if symptoms are triggered by these foods. As studies provide evidence that microscopic colitis is associated with cigarette smoking it is worth advising to stop smoking prior to any drug treatment. When making the decision to suggest a drug treatment to the patient, the individual clinical course of the disease and the severity of diarrhea has to be considered. Present clinical trials report short- and long-term improvement of clinical symptoms like diarrhea and improvement of histological changes. It is not clear what the improvement of histological changes means to the natural course of the disease and whether histological improvement or remission should be treatment goals. Future clinical trials will have to clarify the relevance of histological improvement [94].

Randomized controlled trials have shown that the steroid *budesonide* is an effective treatment for moderate to severe collagenous and lymphocytic colitis. This was confirmed by a recent meta-analysis [95]. Antidiarrheal agents such as loperamide may be used in patients with mild or transient symptoms. Older recommendations for patients with microscopic colitis are based on case reports and, if at all, uncontrolled studies and may be of value if budesonide treatment fails or cannot be tolerated for whatsoever reason.  Table 3 shows an algorithm that may be used in the treatment of patients with microscopic colitis [33].

To date budesonide is the standard treatment for collagenous and lymphocytic colitis. It is advantageous over other steroids like prednisolone due to the high hepatic first pass elimination, which results in the absence of the steroid side effects [95]. Two meta-analyses now confirm that budesonide is highly effective in patients with collagenous an lymphocytic colitis and should be the preferred treatment [95, 96]. A recent retrospective population-based study performed in Olmsted County, Minnesota, USA, identified 80 out of a group of 315 patients with microscopic colitis treated with any steroid between 1986 and 2010. 17 (21%) were treated with prednisone, the remainder patients with budesonide. Chart reviews identified that prednisone-treated patients had a lower response rate of 53% compared to the 83% response rate in the budesonide treatment group. Relapse rate was high for both treatment groups and it was higher in the prednisone treatment group, emphasizing that budesonide is the steroid treatment of first choice in patients with microscopic colitis [75].

Randomized, controlled trials have shown that budesonide therapy (9 mg daily for 6–8 weeks) resulted in a significant improvement in clinical symptoms and significant improvement of histological inflammatory changes [97–101]. The 9 mg daily dose can be given once daily or may be divided up in 3 mg doses and was shown to be effective in the induction of a remission for patients with collagenous and lymphocytic colitis. The overall clinical improvement reported is substantial and rapid and according to the available meta-analyses the overall response is around 80% and the number needed to treat is 2 for collagenous colitis and 3 for lymphocytic colitis [95, 102]. Therefore budesonide is presently the standard treatment of patients with microscopic colitis.

All trials that followed patients after the trial showed a very high rate of relapse within about 2 weeks of budesonide cessation. Relapse can be seen in up to 80% of patients and thus a maintenance therapy is advised. Maintenance therapy in microscopic colitis was studied for up to 26 weeks and a daily dose of 6 mg was able to maintain remission in these patients [95, 103]. Maintenance with budesonide is as high as 80% and the number needed to treat is again 2 for collagenous colitis and not yet studied for lymphocytic colitis, though it would not be expected to be different from the one seen for collagenous colitis [102].

Presently it is unclear how to continue or withdraw treatment following a 26-week period. No studies tested a tapering course of budesonide, but many clinicians employ this over a termination of budesonide in an effort to minimize the likelihood of relapse. Though having limited side effects, long-term use may be associated with steroid side effect and patients on long-term budesonide should be monitored for electrolyte imbalance and high blood sugar, and even a calcium and vitamin D supplementation should be considered.

Other *steroids* like prednisolone were investigated in a double-blind, placebo-controlled randomized trial of oral prednisolone 50 mg daily for 2 weeks in patients with collagenous colitis. Due to the small number of patients included and the short duration of the followup, the results of this trial were inconclusive and of limited value. Presently, steroids other than budesonide in the treatment of microscopic colitis are not supported by evidence from clinical trials [104].

For patients with mild to moderate or just occasional symptoms *antidiarrheal drugs* can be tried though no controlled clinical trials support this treatment. Retrospective trials found some clinical benefit for loperamide treatment with doses ranging from 2 to 16 mg per day [19, 105]. Due to the safety of this agent and the possibility of spontaneous remission of microscopic colitis, loperamide is frequently used for first-line therapy for patients with mild symptoms. But it has to be considered that loperamide was not tested in prospective state-of-the-art clinical trials and thus has to be considered off label. It is furthermore unknown whether loperamide is helpful in long-term treatment of microscopic colitis, and respective trials seem imperative. Whether loperamide has any effect on histological changes remains questionable but from the mode of action it seems unlikely and clinical trials are needed to clarify this point.

Prior to budesonide availability, *aminosalicylates* were the mainstay in microscopic colitis treatment. Aminosalicylate treatment is presently not supported by randomized, plazebo controlled clinical trials. Retrospective case series have suggested symptomatic improvement in up to 50% of patients treated with mesalamine (5-aminosalicylic acid, 5-ASA) [19, 22, 106]. These studies included patients with both subforms, collagenous colitis, and lymphocytic colitis and observation periods ranged up to 6 months. There is only one randomized trial on aminosalicylate use in microscopic colitis and this trial compared mesalamine (800 mg tid) to a combination of mesalamine (800 mg tid) and cholestyramine (4 g/day) in patients with microscopic colitis. Based on clinical and histological outcomes, the combination of mesalamine with cholestyramine was slightly superior [107]. Since response rates in this unfortunately not placebo-controlled trial compare well to the response seen with budesonide treatment, trials comparing budesonide and mesalamine in induction and maintenance of remission are wanted. According to the information given on http://clinicaltrials.gov/, such three armed clinical trials are close to completion [108].

*Other therapies* were either tested in small uncontrolled trials or suggested from retrospective analyses and individual case reports. These approaches include immunosuppressants like azathioprine and methotrexate [109–111], biological anti-TNF treatments [112, 113], *Boswellia serrata* extract, probiotics, pentoxifylline [114], verapamil [115], octreotide, and empirical antibiotic treatment [23, 54, 116, 117]. For probiotics, *E. coli* Nissle 1917 improved stool consistency and stool frequency in an open label study on 14 patients with collagenous colitis, *E. coli* Nissle 1917 was not tested in patients with lymphocytic colitis [118]. Other probiotics like *Lactobacillus acidophilus* LA-5 and *Bifidobacterium animalis* subspp. Lactis B12 failed to be superior to placebo treatment, in a 12-week clinical trial in patients with collagenous colitis [119]. A placebo-controlled, randomized clinical trial testing bismuth subsalicylate in patients with microscopic colitis reported a 100% clinical and histological remission rate in the verum group, compared to 0% in the placebo group [104]. Though reporting a promising approach, due to the limited number of patients included, the study seems to be too preliminary to draw strong conclusions. Though some treatments look promising they cannot be suggested as standard treatments outside of clinical trials. In some patients these medications may be of use due to intolerance or ineffectiveness of the standard medication.

If at any stage drug treatment with budesonide is not sufficient, microscopic colitis should be reconfirmed and differentials should be considered. If this confirms microscopic colitis, the above mentioned drugs may be tried. Newer publications suggest that immunosuppressants like azathioprine or biological anti-TNF treatments should be used but as of present there are no clinical trials testing such an approach [33]. From older publications there are reports of surgical approaches to patients with microscopic colitis but these reports date back into the prebudesonide time and it remains unclear whether this is still needed with powerful drugs being available. Surgical approaches found in literature include subtotal colectomy and diverting ileostomy and in the respective reports these were helpful in individual cases [120, 121].

One important aspect of the treatment of microscopic colitis is the natural course of the disease. There is only limited information available and most of the information dates back to the time before budesonide treatment being available. Thus there is no information on the natural course of untreated patients and therefore our information on the long-term course of the disease is very limited. From the newer budesonide trials it seems that relapse rates are high when medication is stopped. Whether this indicates that most of the patients need life-long medications or whether this indicates that budesonide treatment results in withdrawal symptoms needs to be addressed in future studies. There is presently no such information available from drug trials with other medications and it has to be considered that the trials on other medications were mostly small and uncontrolled studies. One study followed 81 patients with microscopic colitis (44 with lymphocytic colitis and 37 with collagenous colitis) for an average of 7 months. In this observational study patients received different drugs including budesonide and NSAIDs in an uncontrolled fashion. Interestingly the study showed that overall symptom relapse was 30% which is lower than the relapse rate seen in the budesonide trial [122]. The study was too small to observe differences between the drugs used and it is also unclear inasmuch treatment duration drives relapse rates, as the high relapse rates in budesonide relapsers were reported in the short-term clinical trials. What can be taken from this report is that whatever treatment you choose overall response seems to be high and ranges up to 70%. In addition to our lack of knowledge of the clinical long-term course in patients with microscopic colitis we furthermore do not know whether and how the histological changes develop over the course of the disease and whether they fully disappear or whether they can be found lifelong. Thus treatment strategies should follow the information that we have from the controlled clinical trials but should also take into account that the natural course of the disease is unknown ant that careful individual decisions based on the individual patients disease should be made.

For microscopic colitis refractory, intolerant or dependent to steroids, *azathioprine* was used in an uncontrolled fashion in 9 patients. 7 of these patients went into a remission of symptoms and 1 additional patient had at least a significant improvement of symptoms [110]. The patients reported in this retrospective chart analysis were treated with steroids other than budesonide thus it remains unclear whether azathioprine is effective in failures to budesonide. Controlled clinical trials are wanted and seem imperative as with rising incidences of microscopic colitis the number of treatment failures to budesonide are likely to rise as well. Data on methotrexate treatment is conflicting with one uncontrolled trial showing improvement of symptoms in patient with collagenous colitis when given orally and another study showing no symptomatic benefit following s.c. methotrexate in patients that did not respond to oral budesonide [33, 123].

More recently *anti-TNF drugs* were tested in patients refractory to budesonide treatment [112, 113]. One study tested the anti-TNF drug adalimumab in 3 patients who developed side effects or were refractory to methotrexate and budesonide. 2 patients tolerated adalimumab treatment well and were in remission after 6 weeks whereas 1 patient had to stop the medication due to severe side effects [113]. The other study was interested in long-term treatment effects of anti-TNF treatments and reported that within a cohort of 372 patients with microscopic colitis 4 patients received anti-TNF treatment due to failure of success of other medication tried. All 4 received initially infliximab; 3 were later switched to adalimumab due to allergic response or loss of effect. After 1 year 3 patients were still in symptomatic remission documenting that anti-TNF treatment may be an option for patients not responding to other medication [112].

Historical evidence ranging back into the prebudesonide time suggests surgical interventions as an ultima ratio for patients with severe symptoms not responding to the at that time available medication [124]. Subtotal colectomy, sigmoidostomy, or diversion ileostomy was reported in individual cases and it remains doubtful that with budesonide being available, such surgeries still being necessary [33, 121, 125, 126]. Most reports are single reports on ileostomies with largest series reporting less than 10 patients. Interestingly, some reports suggest that the colonic collagenous band can resolve following an ileostomy [121].

## 7. Summary

Microscopic colitis is a common cause of chronic diarrhea especially in older female patients. When other causes of diarrhea like IBS, IBD, or infectious diarrhea are ruled out, a colonoscopy with random biopsies from all regions of the colon and the rectum allows for the diagnosis of either lymphocytic or collagenous colitis. Based on symptom severity and disease duration, a stepwise approach to the treatment is suggested.

## Figures and Tables

**Table 1:** Drugs frequently associated with microscopic colitis.

Aspirin	NSAIDs
Carbamazepine	PPIs
Lisinopril	Madopar
Paroxetine	Flutamide
Sertraline	Ticlopidine
Ranitidine	Acarbose
Simvastatin	Tardyferon
Vinburnine	

**Table 2:** Histopathological features of collagenous colitis and lymphocytic colitis.

Collagenous colitis	Lymphocytic colitis
(1) Thickening of a subepithelial collagen layer of more than 10 um	(1) Intraepithelial lymphocytosis (≥20 IEL per 100 surface epithelial cells)
(2) Inflammation in the lamina propria consisting of mainly lymphocytes and plasma cells	(2) Inflammation in the lamina propria consisting of mainly lymphocytes and plasma cells
(3) Epithelial damage, such as flattening and detachment.	(3) Epithelial damage, such as flattening and detachment
(4) Intraepithelial lymphocytosis (IEL) could be present, but is not necessary for the diagnosis of CC	(4) Subepithelial collagen layer not present or less than <10 um

**Table 3:** Treatment algorithm.

Confirm diagnosis/rule out other disorders
Withdrawal of medications associated with microscopic colitis
Dietary changes (avoid caffeine, lactose)
Trial of loperamide (mild symptoms)
Trial of budesonide (moderate symptoms)
(a) Induction of remission
(b) Maintenance of remission
